# Association of Costs and Days at Home With Transfer Hospital in Home

**DOI:** 10.1001/jamanetworkopen.2021.14920

**Published:** 2021-06-29

**Authors:** Shubing Cai, Orna Intrator, Caitlin Chan, Laurence Buxbaum, Mary Ann Haggerty, Ciaran S. Phibbs, Edna Schwab, Bruce Kinosian

**Affiliations:** 1 Geriatrics and Extended Care Data Analysis Center, Philadelphia, Pennsylvania; 2 Department of Public Health Sciences, University of Rochester, Rochester, New York; 3 VA Palo Alto Health Economics Resource Center, Menlo Park, California; 4 Cpl Michael J. Crescenz VA Medical Center, Philadelphia, Pennsylvania; 5 Department of Medicine, University of Pennsylvania School of Medicine, Philadelphia; 6 Penn Medicine at Home, University of Pennsylvania Health System, Philadelphia; 7 Department of Pediatrics (Neonatal Medicine), Stanford University School of Medicine, Stanford, California; 8 Center for Health Equity Research and Promotion, Philadelphia, Pennsylvania

## Abstract

**Importance:**

New Centers for Medicare & Medicaid Services waivers created a payment mechanism for hospital at home services. Although it is well established that direct admission to hospital at home from the community as a substitute for hospital care provides superior outcomes and lower cost, the effectiveness of transfer hospital at home—that is, completing hospitalization at home—is unclear.

**Objective:**

To evaluate the outcomes of the transfer component of a Veterans Affairs (VA) Hospital in Home program (T-HIH), taking advantage of natural geographical limitations in a program’s service area.

**Design, Setting, and Participants:**

In this quality improvement study, T-HIH was offered to veterans residing in Philadelphia, Pennsylvania, and their outcomes were compared with those of propensity-matched veterans residing in adjacent Camden, New Jersey, who were admitted to the VA hospital from 2012 to 2018. Data analysis was performed from October 2019 to May 2020.

**Intervention:**

Enrollment in the T-HIH program.

**Main Outcomes and Measures:**

The main outcomes were hospital length of stay, 30-day and 90-day readmissions, VA direct costs, combined VA and Medicare costs, mortality, 90-day nursing home use, and days at home after hospital discharge. An intent-to-treat analysis of cost and utilization was performed.

**Results:**

A total of 405 veterans (mean [SD] age, 66.7 [0.83] years; 399 men [98.5%]) with medically complex conditions, primarily congestive heart failure and chronic obstructive pulmonary disease exacerbations (mean [SD] hierarchical condition categories score, 3.54 [0.16]), were enrolled. Ten participants could not be matched, so analyses were performed for 395 veterans (all of whom were men), 98 in the T-HIH group and 297 in the control group. For patients in the T-HIH group compared with the control group, length of stay was 20% lower (6.1 vs 7.7 days; difference, 1.6 days; 95% CI, −3.77 to 0.61 days), VA costs were 20% lower (−$5910; 95% CI, −$13 049 to $1229), combined VA and Medicare costs were 22% lower (−$7002; 95% CI, −$14 314 to $309), readmission rates were similar (23.7% vs 23.0%), the numbers of nursing home days were significantly fewer (0.92 vs 7.45 days; difference, −6.5 days; 95% CI, −12.1 to −0.96 days; *P* = .02), and the number of days at home was 18% higher (81.4 vs 68.8 days; difference, 12.6 days; 95% CI, 3.12 to 22.08 days; *P* = .01).

**Conclusions and Relevance:**

In this study, T-HIH was significantly associated with increased days at home and less nursing home use but was not associated with increased health care system costs.

## Key Points

**Question:**

Is transfer hospital in home (T-HIH) associated with increased days at home without being associated with increased costs?

**Findings:**

In this quality improvement study, T-HIH was significantly associated with 18% more days at home and significantly less posthospital nursing home use but was not associated with increased Veterans Affairs or Medicare costs.

**Meaning:**

These findings suggest that T-HIH provides benefits to patients, payers, and health care systems.

## Teaser

This quality improvement study evaluates the outcomes of transfer hospital in home and its associations with costs and days at home by taking advantage of natural geographical limitations in the program’s service area.

## Introduction

Recent Centers for Medicare & Medicaid Services (CMS) waivers created during the COVID-19 public health emergency have created an opportunity for greatly expanding hospital at home (HaH) services by creating a defined payment mechanism for the program.^1^ Such an expansion is a welcomed addition to proven innovations that have been rapidly scaled during the pandemic, at a time when hospital capacity has been overwhelmed in many areas.^2,3^

HaH is a program that provides hospital-level care outside the hospital, usually at home.^4^ In the US, rigorous observational^5^ and randomized^6^ studies of admission-avoidance HaH, in which patients are admitted directly to HaH, usually from a hospital emergency department, have demonstrated shorter lengths of stay, less delirium, improved function, and lower costs. Less is known about transfer HaH,^3,6,7,8^ where the program facilitates the transfer of patients being treated in the hospital, who require ongoing hospital-level care, to complete their hospital care at home. A challenge for programs is whether the additional costs of hospital-level care at home (and their duration) could outweigh an additional period in the hospital, followed by less-expensive skilled home health care.

In the Veterans Healthcare Administration (VHA), HaH programs are termed Hospital in Home (HIH).^8^ HIH care aligns with high-level VHA strategies to move care to serve veterans effectively in the community and help ease capacity issues in Veterans Affairs (VA) hospitals. The Cpl Michael J Crescenz VA Medical Center (CMCVAMC; Philadelphia, Pennsylvania) has operated a HIH program since 2012, providing both admission-avoidance and transfer HaH care. The program mixes medical management and durable medical equipment (eg, oxygen) from VHA with a bundle of home infusion and home health services purchased from a home health agency, reducing the substantial overhead costs of the typical VHA HIH program, which is fully reliant on VHA personnel. As a result of interstate licensing restrictions, the home health agency providing HIH could not provide services in New Jersey, although a substantial number of veterans served at the CMCVAMC live across the Delaware River in Camden, New Jersey. We took advantage of this geographical restriction to overcome selection bias in evaluating whether the transfer component of the HIH program (T-HIH) was associated with reduced hospital length of stay, readmissions, and posthospital nursing home use, while not being associated with increased VHA or combined VHA and Medicare costs, and whether it was associated with increased days at home in the 90 days after hospital discharge, while not being associated with increased mortality.

## Methods

In this quality improvement study, we identified all veterans receiving T-HIH care from the program’s inception in 2012 through 2018 admitted to the CMCVAMC hospital and matched them with control patients residing in Camden, for whom HIH was unavailable, using methods described in prior admission-avoidance HIH evaluations.^9,10^ Matching variables included demographics (age and age squared, marital status, and VHA priority status P1A, which is given to veterans who had ≥70% service-connected disabilities), hospitalization in the prior 30 days, intensive care unit use during the index hospitalization, number of inpatient diagnoses at admission, JEN Frailty Index score (range, 0-12, with higher scores indicating greater frailty),^11^ the VHA Care Assessment of Need probability of hospitalization or death within 90 days (range, 0%-100%, with higher percentages indicating higher probability of hospitalization or death; the variable is termed pEvent),^12^ diagnosis-related groups (major diagnostic category level), and admission year. We used merged VA and Medicare records for outcome assessment, as described elsewhere.^9,10^ We used the Area Deprivation Index (ADI; range, 0-100, with higher scores indicating greater neighborhood socioeconomic deprivation) from the Neighborhood Atlas to measure social determinants of health.^13^

Outcome measures included costs, utilization, and mortality. We applied an intent-to-treat analysis of cost and utilization, including all veterans from admission to the hospital and followed them through 30-day and 90-day windows. For reporting posthospital utilization outcomes, we restricted the sample to live inpatient discharges, who were capable of having posthospital utilization. Cost outcomes included VA direct costs to provide insight at the health care system level, because direct costs reflect resources that can be recouped, and combined VA and Medicare total costs, because there are substantial post–acute care utilization and costs for dual-eligible Veterans paid by Medicare.

Utilization outcomes included total length of index hospital stay (control patients) or combined index hospital and HIH stays (HIH treated patients) to test whether complementary HIH reduced the number of acute care days. Rehospitalizations were tracked within 30 and 90 days after discharge.

A recently adopted CMS quality measure for their Direct Contracting and Primary Care First models is days at home.^14^ We used a measure of days at home, operationalized as noninstitutional days within 90 days of discharge among live discharges from hospital. Days at home for HIH patients included the days in HIH because these were days not in an institution.

### Statistical Analysis

We conducted a propensity score analysis to estimate the average treatment effect on the treated (ATET) because our target was to evaluate this particular HIH program. ATET compares the average outcomes among the HIH patients and matched controls where all matches within a caliper of 0.01 are selected. Matched controls were selected from all veterans who were hospitalized in CMCVAMC in a given year, who resided in Camden zip codes, with the same major diagnostic category level. This model was estimated using PSMATCH2 in Stata statistical software version 15 (StataCorp). Two-sided *t* tests were used for continuous outcomes. Significance was set at *P* < .05. Standard errors of the estimated ATET were computed from 1000 iterations of bootstrapping the process of generating the propensity score and obtaining the ATET. Data analysis was performed from October 2019 to May 2020.

## Results

A total of 405 veterans (mean [SD] age, 66.7 [0.83] years; 399 men [98.5%]) with medically complex conditions, primarily congestive heart failure and chronic obstructive pulmonary disease exacerbations (mean [SD] hierarchical condition categories score, 3.54 [0.16]), were enrolled. Ten participants could not be matched, primarily because they had greater medical complexity than the available controls, so analyses were performed for 395 veterans (all of whom were men). Among the 395 veterans, 98 were T-HIH patients, of whom 94 were discharged alive from HIH. We found 297 propensity-matched control veterans, of whom 286 were discharged alive from inpatient care. Twenty-three T-HIH patients (21%) living in highly socioeconomically disadvantaged areas could not be matched because there were no control patients living in comparable areas available. Ninety percent of unmatched patients (9 of 10 patients) had a 90-day VHA Care Assessment of Need event probability greater than 0.29 and a mean (SD) hierarchical condition categories score of 4.67 (1.44), with 60% (6 of 10 patients) having a JEN Frailty Index score greater than 7 (highly frail). The analyzed sample had 26.5% (26 patients) having a VHA Care Assessment of Need probability of 90-day hospitalization or death greater than 0.29, mean hierarchical condition categories score of 3.43, and 44% (46 patients) having a JEN Frailty Index score greater than 7. Matching produced balanced cohorts (Table 1). The ADI was higher among T-HIH patients (mean [SD] Philadelphia ADI, 60 [26]) than among control patients (mean [SD] Camden ADI, 35.7 [22]), with 43% of T-HIH patients (42 patients) compared with 11% of control patients (33 patients) having an ADI among the 30% most deprived US Census tracts nationally. More than 19% of Philadelphia patients had ADI scores greater than 85, compared with less than 3% of Camden patients.

**Table 1.** Demographic Characteristics of the Cohorts, Including Those Discharged From Inpatient Hospital to Death

Characteristic	Full sample, patients, No. (%) (n = 405)			Matched sample, patients, % (n = 395)^a^			Unmatched HIH patients, No. (%) (n = 10)
	T-HIH (n = 108)	Controls (n = 297)	Bias, %	T-HIH (n = 98)	Controls (n = 297)	Bias, %	
Age, mean (SD), y	67.7 (1.14)	66.6 (0.72)	9.4	68.2	68.4	−1.9	63.2 (0.16)
Married	28 (25 .9)	120 (40.4)	−31.0	28.6	31.9	−7.2	NA
VHA priority status P1A	42 (38.9)	117 (39.4)	−1.0	38.8	35.2	7.4	4 (40.0)
Hospitalizations in the prior 30 d	13 (12.0)	39 (13.1)	−3.3	13.3	11.9	4.1	NA
Intensive care unit use during index hospitalization	13 (12.0)	62 (20.9)	−23.9	13.3	15.7	−6.5	NA
JEN Frailty Index score (reference, 0-5)							
6	21 (19.4)	57 (19.2)	0.6	19.4	18.8	1.6	2 (20.0)
≥7	52 (48.1)	124 (41.8)	12.8	46.9	44.4	5.2	6 (60.0)
VHA Care Assessment of Need score, probability of an event at 90 d (reference, 90-d event)							
≤0.05	20 (18.5)	84 (28.3)	−23.1	20.4	19.8	1.4	NA
>0.05 to ≤0.29	40 (37.0)	143 (48.1)	−22.5	40.8	41.5	−1.3	NA
>0.29	35 (32.4)	33 (11.1)	53.2	26.5	22.5	10.0	9 (90.0)
Year (reference, 2012-2014)							
2015-2016	43 (42.6)	95 (32.0)	22.0	43.9	41.4	5.1	3 (30.0)
2017-2018	38 (35.2)	60 (20.2)	33.8	31.6	34.6	−6.8	7 (70.0)
Major diagnostic group of the index hospitalization (reference, all other types)							
Respiratory	14 (13.0)	72 (24.2)	−29.2	14.3	15.4	−3.0	NA
Circulatory	80 (74.1)	133 (44.8)	62.3	72.4	73.4	−1.9	9 (90.0)
Hierarchical condition categories score, mean (SD)	3.54 (0.16)	2.79 (0.09)	46.7	3.43	3.36	4.5	4.67 (1.44)
Diagnoses, mean (SD), No.	12.76 (0.45)	10.79 (0.23)	45.1	12.29	12.67	−8.9	17.40 (4.27)

Abbreviations: HIH, Hospital in Home; NA, not applicable; T-HIH, Transfer Hospital in Home; VHA, Veterans Healthcare Administration.

^a^ For the matched sample, SDs are not available for the means and counts are not available for the proportions because we used inverse probability weighting, rather than individual matching.

Table 2 presents the results of the propensity score matched analysis. VA costs were 20% lower at 30 days (−$5910; 95% CI, −$13 049 to $1229) and 13% lower at 90 days (−$5793; 95% CI, −$19 179 to $7594) for HIH patients. Total VA and Medicare costs were 22% lower at 30 days (−$7002; 95% CI, −$14 314 to $309) and 12% lower at 90 days (−$6016; 95% CI, −$20 673 to $83641) for T-HIH patients.

**Table 2.** Results of the Propensity Score–Matched Analysis^a^

Variable	T-HIH	Control	Difference (95% CI)	Bootstrapping SE (*P* value)
Duration of care^b^				
At home, d	81.4	68.8	12.6 (3.12 to 22.08)	4.84 (.009)
At nursing home, d	0.92	7.45	−6.53 (−12.1 to −0.97)	2.84 (.02)
Index hospitalization, d from admission^c^	6.12	7.70	−1.58 (−3.768 to 0.612)	1.12 (.16)
30-d and 90-d outcomes after the discharge (from hospitals for controls, and from HIH discharge for enrollees)^c^				
Readmission rate, %				
30 d	23.5	21.2	2.2 (−11.9 to 16.4)	0.07 (.76)
90 d	35.7	32.2	3.5 (−11.6 to 18.5)	0.08 (.65)
Mortality rate, %				
30 d	4.1	12.5	−8.4 (−16.9 to 0.1)	0.04 (.05)
90 d	5.1	17.6	−12.5 (−23.2 to −1.8)	0.05 (.02)
Mortality (live discharge) restricted to live inpatient discharge, %^b^				
30 d	3.2	5.3	−2.1 (−8.5 to 4.4)	0.03 (.53)
90 d	4.3	10.7	−6.4 (−15.3 to 2.4)	0.05 (.15)
Costs from the inception of the index hospitalization (ie, hospital admission date), $^c^				
VA total cost				
30 d	24 183	30 093	−5910 (−13 049 to 1229)	3643 (.11)
90 d	37 977	43 769	−5793 (−19 179 to 7594)	6830 (.40)
VA and Medicare total cost				
30 d	24 917	31 919	−7002 (−14 314 to 309)	3731 (.06)
90 d	42 270	48 286	−6016 (−20 673 to 8641)	7479 (.42)

Abbreviations: HIH, Hospital in Home; T-HIH, Transfer Hospital in Home; VA, Veteran Affairs.

^a^ For community stays and nursing home stays, the observational window was within 90 d discharge of the index hospitalization. The HIH period was considered as community days. For control patients, we did not include those discharged to death.

^b^ Data are for 286 control patients vs 94 enrollees after matching.

^c^ Data are for 297 control patients vs 98 enrollees after matching.

The hospital length of stay was 20% lower among HIH patients (6.1 vs 7.7 days; difference, 1.6 days; 95% CI, −3.77 to 0.61 days). Overall mortality from admission was lower among the HIH patients at both 30 days (4.1% vs 12.5%) and 90 days (5.1% vs 17.6%). However, when restricted to patients discharged alive, there was no significant difference between the HIH patients and control patients at either 30 days (3.2% vs 5.3%) or 90 days (4.3% vs 10.7%).

There were no differences in 30-day (23.7% vs 23.0%) and 90-day (36.6% vs 34.7%) readmission rates. Numbers of nursing home days in the 90 days following discharge were 88% lower among HIH patients than control patients (0.92 vs 7.45 days; difference, −6.5 days; 95% CI, −12.1 to −0.96 days; *P* = .02). The number of days at home within 90 days following inpatient hospital discharge was 18% higher among HIH patients than control patients (81.4 vs 68.8 days; difference, 12.6 days; 95% CI, 3.12 to 22.08 days; *P* = .01).

## Discussion

By leveraging a geographical restriction on HIH availability, we found that in the small transfer component of a HIH program, nursing home days were decreased by 88% (6.5 days) and days at home were increased by 18% (12.6 days). Mortality and inpatient days were lower in the HIH group, but because of the small sample size, the differences were not significant. Although combined total VA and Medicare costs were not significantly lower among HIH patients than control patients at 30 and 90 days, such costs were unlikely to be increased, allaying concerns that T-HIH would add more costly posthospital days than traditional skilled home nursing would cost.

By taking advantage of the geographical restriction of the HIH program, we were able to reduce the selection bias typical of T-HIH evaluations. We had intended to control for differing levels of neighborhood socioeconomic disadvantage using the ADI but were unable to include ADI in the final propensity model because of the unavailability of highly socioeconomically disadvantaged matched control patients for 23 T-HIH patients (21%) living in highly socioeconomically disadvantaged areas. Not including ADI introduces a conservative bias on the results, given that a greater ADI has been associated with worse health outcomes, including greater readmissions and higher mortality. Compared with less than 3% of Camden patients, more than 19% of Philadelphia patients had ADI scores greater than 85, a level associated with a 9% greater risk of 30-day readmissions.^15^

The greater number of community days among T-HIH patients is an important finding for Accountable Care Organizations and Direct Contracting entities, for whom this is to be a future quality measure. The significantly reduced use of nursing homes after hospitalization^16^ meets a frequent, recent desire by patients, especially at a time when 36% of COVID-19–related deaths are among nursing home residents.^17^

### Limitations

This study has some limitations. The small sample size and large cost variance resulted in lower power for this program evaluation. The referral patterns of physicians at the CMCVAMC leaned toward referring patients with congestive heart failure to T-HIH, thus skewing the diagnostic distribution among HIH patients compared with all hospital admissions. Although the geographical limitation on provision of HIH might have ameliorated the effects of selection bias for T-HIH, making it possible to identify potential control patients for the T-HIH program evaluation, the finding that 21% of T-HIH patients resided in Census tracts with such high ADI scores that not enough potential control patients could be identified prohibited a more complete analysis. Furthermore, the ADI might have inadequately controlled for other geographical differences in outcome among patients residing in Philadelphia and Camden.

## Conclusions

The findings of this quality improvement study suggest that this integrated approach to HaH, with VA contracting for a bundle of home infusion and home nursing services to complete the HIH service menu, can efficiently use existing home health capacity to expand hospital capacity, lower costs, and increase days at home. The new CMS waivers should make this feasible for currently overwhelmed health systems, possibly in partnership with existing community home health agencies.
